# Precipitation and characterization of CaCO3 of *Bacillus amyloliquefaciens* U17 strain producing urease and carbonic anhydrase

**DOI:** 10.3906/biy-1901-56

**Published:** 2019-06-13

**Authors:** Merve TEPE, Şevki ARSLAN, Tamer KORALAY, Nazime MERCAN DOĞAN

**Affiliations:** 1 Department of Biology, Faculty of Science and Arts, Pamukkale University, Denizli, Turkey; 2 Department of Geology, Faculty of Engineering, Pamukkale University, Denizli, Turkey

**Keywords:** Bacillus amyloliquefaciens, biomineralization, carbonic anhydrase, urease, western blotting

## Abstract

In the present study, the properties of calcium carbonate mineralization and urease and carbonic anhydrase activities of *Bacillus amyloliquefaciens* U17 isolated from calcareous soil of Denizli (Turkey) were analyzed. CaCO3 was produced in all growth phases. Strain U17 showed 0.615 ± 0.092 µmol/min/mg urease enzyme activity in calcium mineralization medium and 1.315 ± 0.021 µmol/min/mg urease enzyme activity in Luria-Bertani medium supplemented with urea, whereas it showed 36.03 ± 5.48 nmol/min/mg carbonic anhydrase enzyme activity in CaCO3 precipitation medium and 28.82 ± 3.31 nmol/min/mg carbonic anhydrase enzyme activity in Luria-Bertani medium supplemented with urea. The urease B protein expression level of strain U17 was detected by western blotting for the first time. The produced CaCO3 crystals were analyzed by X-ray diffraction, X-ray fluorescence, confocal RAMAN spectrophotometer, scanning electron microscopy, and electron probe microanalyzer for the evaluation of their morphological and elemental properties. Rhombohedral vaterite and layered calcite crystals were clearly detected and verified by mineralogical analyses. All these results showed that strain U17 can be used in many engineering and geological applications due to its CaCO3 precipitation ability.

## 1. Introduction

Biomineralization is the process of synthesis of mineral materials by organisms. It is commonly acknowledged that prokaryotes are more capable than other organisms in biomineralization processing (Zavarzin, 2002). One of the well-known examples of bacterial biomineralization is calcium carbonate (CaCO3) precipitation and it is well established that many bacteria have the capability of producing various forms of CaCO3 in a different environment. Although anhydrous polymorphs such as calcite, aragonite, and vaterite and hydrated crystalline forms such as monohydrocalcite and ikaite are produced as a result of the biomineralization of CaCO3 (Rieger et al., 2007; Gower, 2008; Gebauer et al., 2010), calcite and vaterite are the most common forms of CaCO3 (Rodriguez-Navarro et al., 2007; González-Muñoz et al., 2010). Moreover, these anhydrous polymorphs are different. For example, although vaterite is thermodynamically stable with respect to amorphous calcium carbonate, it is metastable with regard to aragonite and calcite. Furthermore, while vaterite is rarely found in geological settings, it is a fundamental precursor in many of the carbonate formation processes (Wang and Becker, 2009). Microorganisms and minerals interact on all time and spatial scales. Microbiologically induced calcium carbonate precipitation (MICCP) can be given as a good example of this kind of interaction. MICCP is produced by the metabolic activities of bacteria such as photosynthesis, nitrate or sulfate reduction, urea hydrolysis, and other similar activities that cause the precipitation of CaCO3 (Benzerara et al., 2011). In MICCP, which is governed by soil bacteria specifically, cellular enzyme activities (especially urease and carbonic anhydrase) affect the chemical conditions of the environment to promote mineralization (Hammes and Verstraete, 2002). Therefore, it is possible to promote MICCP by managing the growth conditions and enzyme activity of the microorganisms and the saturation of the environment for the proposed ureolytically driven MICCP engineering applications. MICCP has important applications in many problems such as the fixation of metal contamination in soil and water, the strengthening of sand, and the enhancement of the strength of cement (Phillips et al., 2013).Although there are different important applications of biomineralization of CaCO3, few bacterial strains have been studied in terms of its potential. In this regard, the aim of this research is to determine and to develop the MICCP potential and abilities to produce urease (UA) and carbonic anhydrase (CA) enzymes of our local soil bacterium, *Bacillus amyloliquefaciens *U17, extracted from calcareous soil of Denizli, Turkey. The morphological and elemental properties of the CaCO3 crystals were also analyzed in this study.

## 2. Materials and methods

### 2.1. Bacterial strain and determination of amount of CaCO3 in growth medium

The bacterial strain was identified by 16S rDNA gene analysis at the Life Sciences Research and Application Center of Gazi University (Ankara, Turkey) and deposited in the Bacteriology Laboratory, Department of Biology at Pamukkale University, Denizli, Turkey. In order to determine the growth rate of strain U17, bacterial cultures diluted with sterile saline-water (10-1 to 10-10) were inoculated on nutrient agar plates (100 µL) for each mineralization and the number of colony forming units (CFUs) was calculated after incubation at 37 °C for 24 h. Calcium precipitation medium (CPM) was used for CaCO3 precipitation (Ferris et al., 1996; Whiffin et al., 2007). Urea concentration was 25 mM, the initial pH was adjusted to 6.5, growth temperature was 37 °C, and inoculation rate was 10%. The amount of CaCO3 was determined by EDTA titrimetric method and calculated by the formula of [CaCO3 = (V1 × M × 1000)/V2)], where V1 is consumed EDTA, M is 1 mL of EDTA = 0.96 mg CaCO3, and V2 is sample amount (mL) (APHA, 1989). 

### 2.2. Enzyme activity determination studies

#### 2.2.1. Monitoring urease and carbonic anhydrase activity

Urea agar was used for screening the ureolytic activity of U17. The plates were prepared following Christensen’s 1946 procedure. Color change in the medium from orange to pink occurred after 48 h of incubation at 37 °C, indicating the hydrolysis of urea by U17. Differential detection of CA activity was made on tryptic soy agar plates with the method improved by Ramanan et al. 2009. After colonies appeared on plates, 10 mM p-nitrophenyl acetate (p-NPA) solution was sprayed onto solid agar. Color change to bright yellow occurred in the presence of CA enzyme activity as a result of the degradation of p-NPA to *p*-nitrophenol and acetate.

#### 2.2.2. Analytic methods

The U17 strain was grown on either LB-Miller medium supplemented with 25 mM sterilized urea (LB-urea) or CPM. Cultures were incubated at 37 °C with shaking overnight. Cells were harvested by centrifugation at 6000 rpm for 20 min. The pellet was suspended in 2 mL of lysis buffer and sonicated on ice for 30-s pulses for a total period of 5 min. Total protein concentration of the crude enzyme extract was determined by the method of Lowry et al. 1951. Bovine serum albumin was used as the standard.

#### 2.2.3. Urease enzyme activity

UA enzyme activity was determined by measuring ammonia by using a phenol-hypochlorite reaction with slight modifications (Weatherburn, 1967). The crude enzyme extracts from U17 grown on two different media were used for measurement of UA activity. The crude enzyme (100 µL) was added to 500 µL of reaction buffer (100 mM KPi buffer, pH 8.0, containing 50 mM urea). This mixture was incubated at 37 °C for 30 min. Then 50 µL of this mixture was added to 500 µL of phenol-nitroprusside solution, and 500 µL of alkaline sodium hypochlorite solution was added and color development was monitored at room temperature for 30 min. Absorbance was measured at room temperature at 630 nm. An ammonium sulfate standard (5-50 µM) was used for calculation of urease enzyme activity. 

#### 2.2.4. Carbonic anhydrase activity

CA activity was determined by measuring the amount of *p*-nitrophenol according to the method described by Armstrong et al. 1966, with a slight modification. Briefly, 100 µL of crude enzyme was added to 900 µL of reaction mixture containing phosphate buffer (100 mM, pH 7.2) and *p*-nitrophenyl acetate (3 mM). The color change was measured at 412 nm for at least 5 min and the *p*-nitrophenol standard curve was used for calculation of the enzyme activity. 

### 2.3. Western blotting 

SDS-PAGE and western blotting were performed as described previously (Arınç et al., 2007). In this method, 75 µg of sonicated bacterial protein was separated on 8% polyacrylamide gels using a discontinuous buffer system (Laemmli, 1970). Separated proteins were then transferred from gel to a nitrocellulose membrane by using a Trans-Blot electrophoretic transfer cell (Bio-Rad) containing Tris-glycine buffer, pH 8.3, and ethanol at 90 V. Immunochemical staining of the separated proteins on the nitrocellulose sheet was done by using the 1/1000 diluted Abcam anti-*Helicobacter pylori* urease antibody 127916 in Tris-buffered saline plus 0.05% Tween 20 (TBST) containing 5% nonfat dried milk. The blot was further incubated with secondary antibodies and alkaline phosphatase conjugated goat antirabbit IgG (diluted 1/5000 with TBST) for 1 h. Alkaline phosphatase activity was detected as described by Ey and Ashman 1986. The final images were photographed and protein bands were quantified using Scion Image Version Beta 4.0.2 software.

### 2.4. Mineralogical analysis

In order to collect CaCO3 minerals obtained from the U17 strain, cell culture was centrifuged (NuveNF 400R) and freeze-dried (Telstar Lyoquest Freeze-Dryer). X-ray diffraction (XRD), X-ray fluorescence (XRF), confocal RAMAN spectrophotometer (CRS), and electron probe microanalyzer (EPMA) analyses were executed at the Ankara University Earth Sciences Application and Research Center (YEBİM, Ankara, Turkey). The qualitative mineralogical composition of the CaCO3 samples was determined with XRD, using an Inel Equinox 1000 diffractometer (instrumental conditions: CoKα radiation obtained at 30 kV and 30 Ma, 5-80° 2θ investigation range, 0.030° step).Elemental compositions of the powder samples were analyzed by a Spectro XLAB2000 XRF spectrometer. The instrumentation was equipped with a 400-W Rh end-window tube and Si (Li) detector with a resolution of 148 eV (1000 cps Mn Kα). The available targets were Al2O3 and B4C used as a Barkla polarizer, highly oriented pyrolytic graphite (HOPG)-crystal used as a Bragg polarizer, and Al, Mo, and Co used as secondary targets. The irradiation chamber was operated under a vacuum system. The****CRS technique was applied on powder thin sections. Raman spectra (100-1200 cm-1) were obtained using a Thermo-DXR Raman spectrometer. This spectrometer has the 785-nm excitation of air-cooled argon lasers. The aperture is a 25-µm slit, the grating has estimated resolution of 600 lines/mm, and the spot sizes are 2.6-4.4 cm-1 and 0.7 µm, respectively. EPMA analyses were performed to determine the mineral composition of powder samples. All analyses were made on carbon coated samples using a JEOL JXA-8230 SuperProbe microscope. Operating conditions for quantitative analyses were 15 kV accelerating voltage and 20 nA beam current. Natural mineral standards and the ZAF matrix correction routine were used. Finally,****SEM analysis was conducted with a ZEISS-LEO 1430 scanning electron microscope with accelerating voltage of 15 kV at the Akdeniz University Medical Faculty’s Electron Microscopy Image Analysis Unit (TEMGA, Antalya, Turkey). 

## 3. Results

### 3.1. Bacterial strain and growth conditions

**Figure 1:**
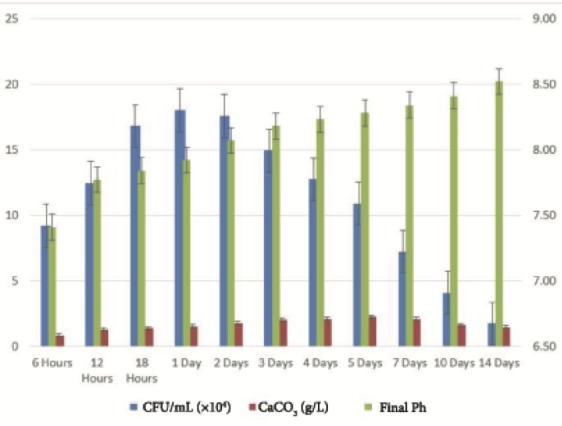
CaCO3 mineralization and growth scheme of *Bacillus amyloliquefaciens *U17 (g/L CaCO3) and pH change throughout incubation period (25 mM urea, 25 mM CaCl2, 25 mM NaHCO3, 10% inoculation rate, t = 37 °C, pHi: 6.50).

The bacterial strain was identified by 16S rDNA analysis at the Life Sciences Research and Application Center of Gazi University (Ankara, Turkey). A product of 1338 bp was sequenced and blasted by using the NCBI BLAST database. According to that result, the U17 strain was 100% identical to *Bacillus amyloliquefaciens*******(GenBank Accession Number MK878414). Moreover, this strain was stocked in the Refik Saydam National Type Culture Collection (RSKK) with code number RSKK 19001.The U17 strain started CaCO3 mineralization within the first 6 h of incubation (0.828 ± 0.020 g/L CaCO3) and mineralization gradually increased until the fifth day of incubation (2.248 ± 0.011 g/L CaCO3). After 5 days, mineralization continued to decrease gradually. The incubation period was started with CPM adjusted to initial pH 6.50 and slight changes in pH occurred after incubation (pH 7.41 for 6 h), and it kept increasing throughout the 14 days of the incubation period. After the highest rate of precipitation (pH 8.28 for 5 days), the CaCO3 amount was detected to diminish, whereas pH continued to increase up to the end of the incubation period (Figure  1).

### 3.2. Urease and carbonic anhydrase activities and western blotting

**Figure 2:**
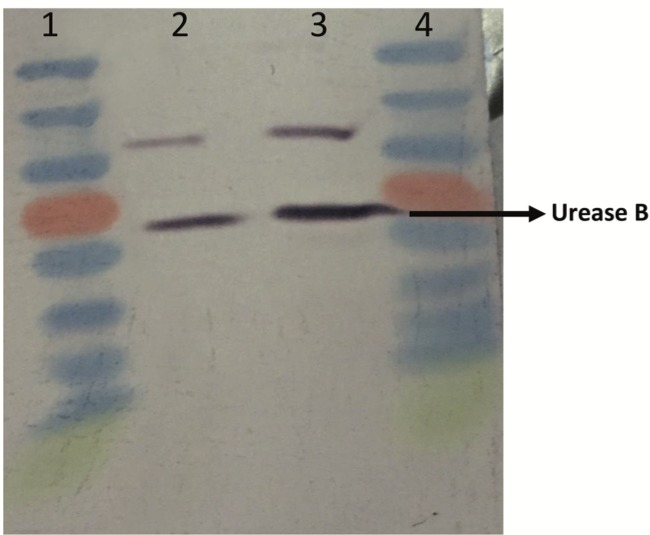
Effects of different bacterial media on urease B protein expression. *Bacillus amyloliquefaciens* U17 was grown on CPM and LBurea medium. Protein bands were detected by western blot analysis and quantitated by densitometry. Lanes: 1, molecular mass standard (protein ruler); 2, U17 urease B protein in LB-urea medium; 3, U17 urease B protein in CPM; 4, molecular mass standard (protein ruler).

The UA enzyme activity of U17 was calculated as 0.615 ± 0.092 µmol/min/mg in CPM and 1.315 ± 0.021 µmol/min/mg in LB-urea medium. UA enzyme activity was induced approximately 2-fold in LB-urea medium compared to CPM. In addition to UA enzyme activity, the CA activity of U17 was determined throughout this study. This enzyme activity was found as 36.03 ± 5.48 nmol/min/mg in CPM. On the other hand, CA activity was calculated as 28.82 ± 3.31 nmol/min/mg in LB-urea medium (Table 1). The U17 urease protein level was identified by immunochemical detection on western blots in this study by utilizing polyclonal antibodies raised against *Helicobacter pylo*ri urease (Figure  2). An increase in the staining intensity of the immunoreactive band of urease from strain U17 grown in LB-urea with respect to CPM-grown cells was observed in the current study. Densitometric scanning of western blot results showed that an approximately 5-fold difference was observed between the two media.

**Table 1:** Urease and carbonic anhydrase enzyme activities of Bacillus amyloliquefaciens U17.

Urease enzyme activity (µmol/min/mg)		Carbonic anhydrase enzyme activity (nmol/min/mg)	
CPM	LB-Urea	CPM	LB-Urea
0.615 ± 0.092	1.315 ± 0.021	36.03 ± 5.48	28.82 ± 3.31

### 3.3. Mineralogical evaluations

**Figure 3:**
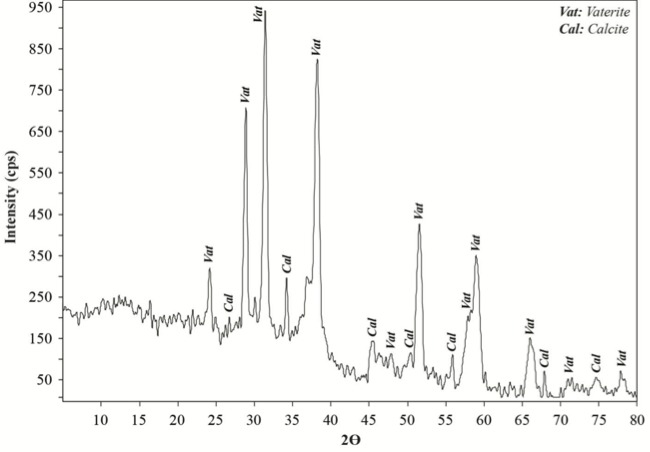
XRD pattern of CaCO3 precipitated by Bacillus amyloliquefaciens U17.

**Figure 4:**
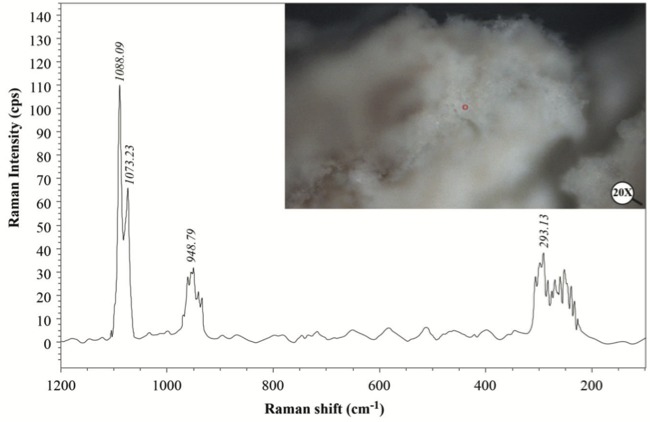
CRS analysis of CaCO3 produced by Bacillus amyloliquefaciens U17.

**Figure 5:**
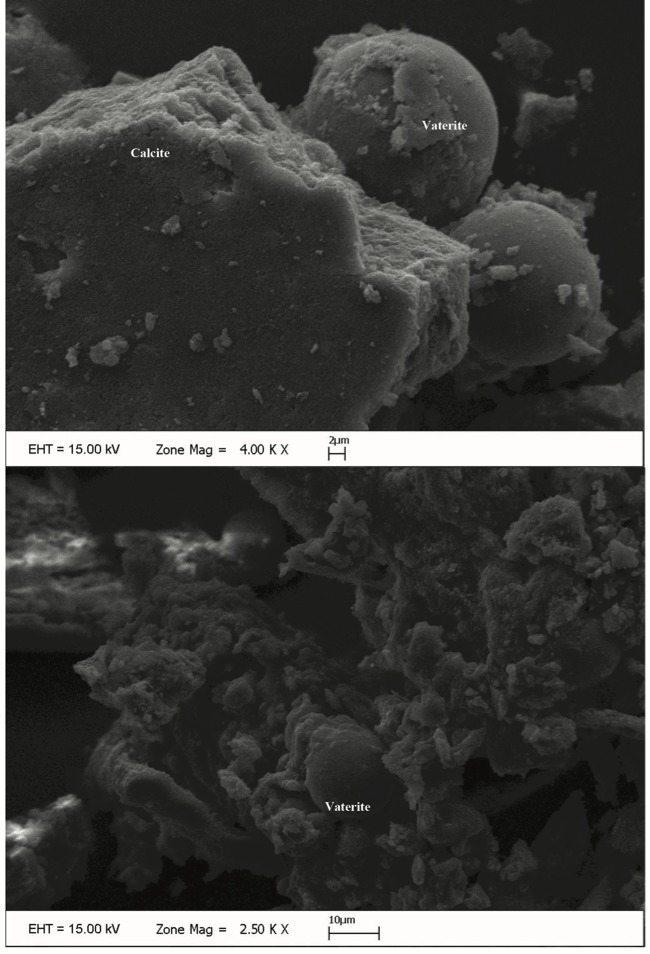
SEM images of CaCO3 precipitated by Bacillus amyloliquefaciens U17.

**Figure 6:**
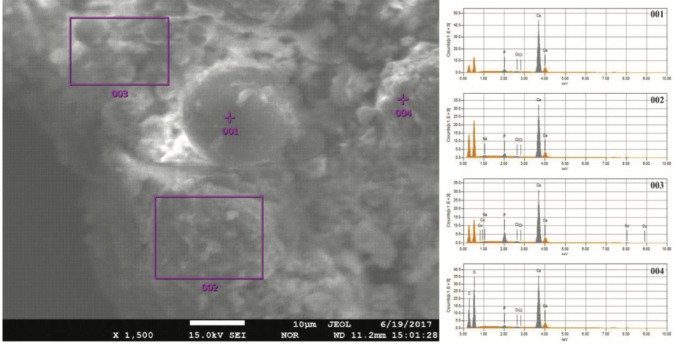
EPMA analysis of CaCO3 precipitated by Bacillus amyloliquefaciens U17.

**Figure 7:**
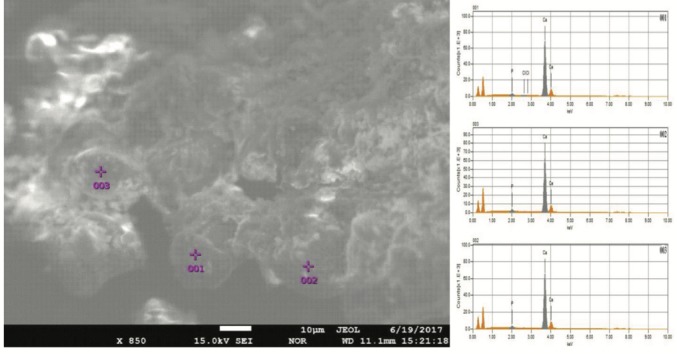
EPMA analysis of CaCO3 produced by Bacillus amyloliquefaciens U17 from another part of the same sample.

We performed XRD, XRF, CRS, SEM, and EPMA analyses in order to validate the mineral structure on the same powder sample produced by the U17 strain in CPM. Characteristic calcite and vaterite peaks were observed in XRD analysis****(Figure  3). Si (0.1598%), P (3.9%), Cl (1.26%), Ca (29.25%), Mg (0.011%), Zn (242.2 ppm), Sr (11.2 ppm), Zr (10.3 ppm), Ba (13.9 ppm), and U (17.7 ppm) elements were recorded to differ slightly compared to other elements in XRF analysis. CRS analysis of the powder sample produced by the U17 strain showed unique CaCO3 peaks with respect to Raman shifts (Figure  4). As a result of SEM analysis, we clearly observed calcite and vaterite minerals (Figure  5). EPMA qualitative analyses from two different parts of the same sample (Figures 6 and 7) also showed structures related to CaCO3 and rhombohedral vaterite crystals were clearly observed. Point and area analysis results (Tables 2 and 3) showed a high rate of Ca element and a particularly low rate of Cl. It proved the degradation of CaCl2 and transformation to CaCO3 crystals with the enzyme activity of U17. Low rates of Na, P, and Cu were detected in the biofilm structure and Na and P were thought to originate from the bacterium’s own genetic structure, while Cu was thought to originate from the structure of MICCP enzymes. 

**Table 2:** EPMA quantitative analysis of CaCO3 produced by Bacillus amyloliquefaciens U17 (data belonging to Figure 6)

Formula	Spot No. 001		Region No. 002		Region No. 003		Spot No. 004	
	Mass %	Atom %	Mass %	Atom %	Mass %	Atom %	Mass %	Atom %
Ca	96.04	95.05	94.65	93.02	85.80	83.18	97.47	96.81
Cl	0.70	0.79	0.69	0.76	1.02	1.12	0.32	0.36
P	3.25	4.17	4.01	5.11	11.08	13.90	2.21	2.84
Na	-	-	0.65	1.11	0.48	0.81	-	-
Cu	-	-	-	-	1.62	0.99	-	-
Total	100.00	100.00	100.00	100.00	100.00	100.00	100.00	100.00

**Table 3:** Quantitative EPMA analysis of produced CaCO3 by Bacillus amyloliquefaciens U17 from another part of the same sample (data belonging to Figure 7).

Formula	Spot No. 001		Spot No. 002		Spot No. 003		Mass %	Atom %	Mass %	Atom %	Mass %	Atom %
Ca	98.12	97.65	98.40	97.94	95.77	94.60
Cl	0.27	0.31	-	-	-	-
P	1.58	2.04	1.60	2.06	4.23	5.40
Total	100.00	100.00	100.00	100.00	100.00	100.00

## 4. Discussion

Ureolytic bacteria precipitate the excess of calcium as CaCO3 in their environments. That bacterial metabolic event has an important role in many processes including geological and engineering applications such as stone conservations and sandy soil improvement (Navarro et al., 2012; Akyol et al., 2017). Our previous studies demonstrated that increasing incubation time is very effective in transforming vaterite crystals to calcite, and *Paenibacillus favisporus* U3 isolated from Denizli (Turkey) had the maximum CaCO3 precipitation among the tested strains and it was utilized for geotechnical studies (Akyol et al., 2017). In this study, we researched the ability of bacterial CaCO3 production by another isolate, *B. amyloliquefaciens *U17, from Denizli. The UA and CA activities of strain U17 were also determined, and we showed the UA enzyme band by western blotting. Moreover, the obtained CaCO3 product was studied by SEM, XRD, CRS, and EPMA methods. CaCO3 was precipitated by *B. amyloliquefaciens* strain U17 in mineralization conditions (urea concentration = 25 mM, pHinitial = 6.5, pHfinal = 8.28, temperature = 37 °C, inoculation rate = 10%) as given in Figure  1. The optimum CaCO3 production was observed in the first 5 days. The maximum CaCO3 amount was recorded at 5 days of incubation as 2.248 g/L. The amount of CaCO3 decreased according to incubation times. In our previous study, *P. favisporus* U3 produced the maximum amount of CaCO3 (2.805 g/L) after the 10th day of incubation (Akyol et al., 2017). This result revealed that the effect of elevated incubation time on CaCO3 contents has great importance for CaCO3 production, and these properties depend on the strain and varies from strain to strain. As is known, microbial CaCO3 precipitation occurs in alkaline environments (Stocks-Fischer et al., 1999; Okwadha and Li, 2010; Qian et al., 2010). In our experiment, the final pH in maximum mineralization time (5 days) was also 8.28. The increasing of pH caused urea hydrolysis in precipitation medium (Figure  1). In our study, we also evaluated the relationship between cell growth and amount of CaCO3 in culture medium. For this reason, cell growth was measured as CFUs. We did not utilize measurements of optical density due to the CaCO3 crystals suspended in the culture medium. The bacterial population was 9.21 × 104 to 18.02 × 104 CFU/mL in the course of cultivation. The maximum growth rate was 18.02 × 104 cfu/mL after 24 h of cultivation and the amount of precipitated CaCO3 was 1.528 ± 0.013 g/L after 24 h of cultivation. After 14 days the growth rate finally decreased to 1.73 × 104 cfu/mL, while mineralization of CaCO3 continued (1.477 ± 0.101 g/L) during the whole 14 days of cultivation. Calcium mineralization started 6 h after the first inoculum and continued to 14 days of incubation. Although the number of cells in the first 6-h period (9.21 × 104 cells) was low, the amount of urease and carbonic anhydrase enzymes released from the cells might have been enough for calcium mineralization (Figure  1). Increases in the amount of CaCO3 and the number of cells were observed during 4 days of incubation. We considered that the number of cells, the amount of enzyme, and the pH of the growth medium were suitable for calcium mineralization at the 4th day. Although cell concentration started to decline at the 5th day of incubation, the amount of calcium carbonate reached a maximum. After that day, we observed a decline in cell concentration and calcium carbonate mineralization. These decreases might be due to the amount of enzyme released by cell, the amount of calcium, and the more alkali pH of the medium. According to a study by Tirkolaei and Bilsel 2017, many factors such as initial cell concentration, initial calcium and urea concentration, and temperature can affect the precipitation rate and maximum amount of precipitation. Therefore, we observed different cell numbers and different precipitation rates on different days in this study.UA and CA are two main enzymes involved in CaCO3 precipitation. A recent study showed that precipitation of calcium carbonate was significantly reduced when both enzymes were inhibited separately by specific inhibitors (Dhami et al., 2014). As can be understood in this study, both enzymes are crucial for efficient mineralization and work synergistically, but they have different role in this process. UA hydrolyses urea into ammonium and carbonate and helps in keeping the alkaline pH, which promotes the calcification process. On the other hand, CA is involved in the reversible hydration of CO2 (Dhami et al., 2014). In the present study, *B. amyloliquefaciens* U17 showing high CaCO3 precipitation was tested for its UA and CA activity in two different media. Results showed that U17 had high UA and CA activity (Table 1). Similar to our results, many bacterial strains including *Bacillus* and *Pseudomonas* with high enzyme activities showed high CaCO3 precipitation (Dhami et al., 2014; Priya and Kannan, 2017). In addition to these results, it has been shown for the first time in our study that these enzyme activities changed in different media. UA enzyme activity was regulated by environmental conditions such as nitrogen content. The nitrogen amount of CPM and LB was different. Therefore, enzyme activity was different in the two media. This result was also confirmed by western blot studies. One of the subunits of UA, namely urease B, was induced in CPM compared to LB medium. Future work is needed to resolve underlying mechanisms of UA induction.XRD is a rapid analytical technique used for identification of various materials from microorganisms. It has been shown previously that calcite and vaterite are major crystal types in bacterial CaCO3 deposits (Daskalakis et al., 2015; Vahabi et al., 2015; Seifan et al., 2016; Akyol et al., 2017). In the current study, the XRD analysis of the bacterial precipitate showed that the precipitate contained both vaterite and calcite minerals (Figure  3). Moreover, the vaterite and calcite crystals in the CaCO3 precipitate obtained from U17 were clearly observed by SEM analysis. Bergdale et al. 2012 and Okyay et al. 2014 reported that calcium carbonate crystals were rhombohedral structures that aggregated into spherical and semispherical structures. As can be seen in Figure  5similar surface morphologies and similar shapes were observed in our study. Dhami et al. 2013 and Mudgil et al. 2018 also indicated that different bacteria species produced different variations of crystals, such as vaterite, calcite, and jungite. The EPMA analysis also revealed that the element present in the highest amount in the samples was Ca. On the other hand, results of the detailed analysis of the present bacterial CaCO3 samples were somewhat similar to those for previously reported crystal types from different bacteria species (Akyol et al., 2017), which confirmed that vaterite and calcite are the most common mineral types in bacteria. Vaterite in particular has been extensively synthesized in culture media. In our experiments, the vaterite ratio of bacterial CaCO3 obtained from U17 was higher than the calcite ratio. This result was in agreement with the previously published data of some scientific papers (Rodriguez-Navarro et al., 2007; Achal and Pan, 2014; Akyol et al., 2017). It is well known that the morphology, composition, and orientation of biocrystals are dependent on the features of the microbial species and the presence of urea (Wang and Nilsen-Hamilton, 2013; Otlewska and Gutarowska, 2016). Moreover, a high ratio of vaterite is advantageous in some engineering applications such as bioconcrete. According to Seifan et al. 2017, vaterite can occupy more space in bioconcrete than calcite due to its lower density. This increases the performance of the bioconcrete. Raman microscopy analysis performed in the present study provides further evidence on the mineralogy of CaCO3 produced by the U17 strain. The peak at 1088.09 cm-1 was predominant (Figure  4). This result agrees well with the study by Bai et al. 2017, who reported that the most notable peak was at 1088 cm-1, which was caused by the symmetric stretching vibration of the internal carbonate ion. According to the XRF analysis, the high calcium ratio in bacterial CaCO3 produced by strain U17 verified this finding. Similarly, XRF element analysis in our experiments supported this finding. Si and P elements were correlated with the bacterium’s own genetic structure and similarly the Cu and Zn levels were correlated with bacterial enzymes. Zn was correlated with the structure of the UA and CA enzymes. Mg, U, Sr, and Ba elements are known to have a role in CaCO3 formation process (Sano et al., 2005). In conclusion, in the present study, the mineralization of CaCO3 and the UA and CA enzyme activities of *Bacillus amyloliquefaciens* strain U17 have been investigated for the first time. In general, it was shown that precipitation of CaCO3 was initiated at the beginning of the cultivation and the amount of CaCO3 increased during the exponential growth phase. Moreover, the mineralization of CaCO3 was shown to continue in the stationary and death phases. The bacterial CaCO3 products were analyzed by XRD, XRF, CRS, and EPMA. Vaterite was observed to be dominant, with minor calcite. In addition to these results, the specific activities of UA and CA and UA protein level in two different media were determined. All of these results showed that *Bacillus* *amyloliquefaciens *strain U17 may have potential applications in many geological and engineering processes including strengthening sand and enhancing the strength of cement.

## Acknowledgment 

This research was financially supported by the Pamukkale University Scientific Research Projects Coordination Department (Project Number: 2017FEBE016).

## References

[ref1] (2014). Influence of calcium sources on microbially induced calcium carbonate precipitation by Bacillus sp. CR2. Applied Biochemistry and Biotechnology.

[ref2] (2017). Strengthening sandy soils by microbial methods. Arabian Journal of Geosciences.

[ref3] (1989). Standard Methods for the Examination of Water and Wastewater, 17th Edition. American Public Health Association.

[ref4] (2007). Effects of diabetes on rabbit kidney and lung CYP2E1 and CYP2B4 expression and drug metabolism and potentiation of carcinogenic activity of N-nitrosodimethylamine in kidney and lung. Food and Chemical Toxicology.

[ref5] (1966). Purification and properties of human erythrocyte carbonic anhydrases. Journal of Biological Chemistry.

[ref6] (2017). Experimental and visual research on the microbial induced carbonate precipitation by Pseudomonas aeruginosa. AMB Express.

[ref7] (2012). Engineered biosealant strains producing inorganic and organic biopolymers. Journal of Biotechnology.

[ref8] (2011). Significance, mechanisms and environmental implications of microbial biomineralization. Comptes Rendus Geoscience.

[ref9] (1946). Urea decomposition as a means of differentiating Proteus and paracolon cultures from each other and from Salmonella and Shigella types. Journal of Bacteriology.

[ref10] (2015). Vaterite bio-precipitation induced by Bacillus pumilus isolated from a solutional cave in Paiania. International Biodeterioration & Biodegradation.

[ref11] (2013). Bacillus megaterium mediated mineralization of calcium carbonate as biogenic surface treatment of green building materials. World Journal of Microbiology and Biotechnology.

[ref12] (2014). Synergistic role of bacterial urease and carbonic anhydrase in carbonate mineralization. Applied Biochemistry and Biotechnology.

[ref13] (1986). The use of alkaline phosphatase-conjugated anti-immunoglobulin with immunoblots for determining the specificity of monoclonal antibodies to protein mixtures. Methods in Enzymology.

[ref14] (1996). Bacteriogenic mineral plugging. Journal of Canadian Petroleum Technology.

[ref15] (2010). Proto‐calcite and proto‐vaterite in amorphous calcium carbonates.

[ref16] (2010). Bacterial biomineralization: new insights from Myxococcus-induced mineral precipitation.

[ref17] (2008). Biomimetic model systems for investigating the amorphous precursor pathway and its role in biomineralization. Chemical Reviews.

[ref18] (2002). Key roles of pH and calcium metabolism in microbial carbonate precipitation. Reviews in Environmental Science and Biotechnology.

[ref19] (1970). Cleavage of structural proteins during the assembly of the head of bacteriophage T4. Nature.

[ref20] (1951). Protein measurement with the Folin phenol reagent. Journal of Biological Chemistry.

[ref21] (2018). Biomineralization potential of Bacillus subtilis, Rummeliibacillus stabekisii and Staphylococcus epidermidis strains in vitro isolated from Speleothems, Khasi Hill Caves. Geomicrobiology Journal.

[ref22] (2012). When structure means conservation: effect of aggregate structure in controlling microbial responses to rewetting events. Soil Biology and Biochemistry.

[ref23] (2010). Optimum conditions for microbial carbonate precipitation. Chemosphere.

[ref24] (2014). Optimized carbonate micro-particle production by Sporosarcina pasteurii using response surface methodology. Ecological Engineering.

[ref25] (2016). Environmental parameters conditioning microbially induced mineralization under the experimental model conditions. Acta Biochimica Polonica.

[ref26] (2013). Engineered applications of ureolytic biomineralization: a review.

[ref27] (2017). Effect of carbonic anhydrase and urease on bacterial calcium carbonate precipitation. International Journal of Pharma and Bio Sciences.

[ref28] (2010). Theory of microbial carbonate precipitation and its application in restoration of cement-based materials defects. Chinese Journal of Chemistry.

[ref29] (2009). Purification and characterization of a novel plant-type carbonic anhydrase from Bacillus subtilis. Biotechnology and Bioprocess Engineering.

[ref30] (2007). Precursor structures in the crystallization/precipitation processes of CaCO3 and control of particle formation by polyelectrolytes.

[ref31] (2007). Bacterially mediated mineralization of vaterite. Geochimica et Cosmochimica Acta.

[ref32] (2005). Nano-SIMS analysis of Mg, Sr, Ba and U in natural calcium carbonate.

[ref33] (2016). Induced calcium carbonate precipitation using Bacillus species. Applied Microbiology and Biotechnology.

[ref34] (1999). Microbiological precipitation of CaCO3. Soil Biology and Biochemistry.

[ref35] (2017). Estimation on ureolysis-based microbially induced calcium carbonate precipitation progress for geotechnical applications. Marine Georesources & Geotechnology.

[ref36] (2015). Calcium carbonate precipitation by strain Bacillus licheniformis AK01, newly isolated from loamy soil: a promising alternative for sealing cement‐based materials. Journal of Basic Microbiology.

[ref37] (2009). Structure and carbonate orientation of vaterite (CaCO3). American Mineralogist.

[ref38] (2013). Biomineralization proteins: from vertebrates to bacteria. Frontiers in Biology.

[ref39] (1967). Phenol-hypochlorite reaction for determination of ammonia. Analytical Chemistry.

[ref40] (2007). Microbial carbonate precipitation as a soil improvement technique. Geomicrobiology Journal.

[ref41] (2002). Microbial geochemical calcium cycle. Microbiology.

